# Mineral solubilizing microorganisms and their combination with plants enhance slope stability by regulating soil aggregate structure

**DOI:** 10.3389/fpls.2023.1303102

**Published:** 2023-12-28

**Authors:** Lingjian Wang, Xinggang Tang, Xin Liu, Rengui Xue, Jinchi Zhang

**Affiliations:** ^1^ Jiangsu Province Key Laboratory of Soil and Water Conservation and Ecological Restoration, Nanjing Forestry University, Nanjing, Jiangsu, China; ^2^ Jiangxi Institute of Land Space Survey and Planning, Nanchang, Jiangxi, China; ^3^ Technology Innovation Center for Land Spatial Eco-protection and Restoration in Great Lakes Basin, Ministry of Natural Resources (MNR), Nanchang, Jiangxi, China

**Keywords:** slope stability, organic matter characteristics, infrared spectral analysis, soil aggregates, structural equation modeling

## Abstract

**Introduction:**

The stability of exposed slopes is prone to natural disasters, seriously threatening socio-economic and human security. Through years of exploration and research, we proposed an active permanent greening (APG) method based on patented mineral solubilizing microorganisms (MSMs) as an improvement over the traditional greening method.

**Methods:**

In this study, we selected two MSMs (*Bacillus thuringiensis* and *Gongronella butleri*) and a plant species (*Lolium perenne* L.) set up six treatments (T1, T2, T3, T4, T5, and T6) to investigate the effectiveness of the MSMs and their combinations with the plant species on the soil stability using APG method.

**Results:**

We noted that both MSMs and the plant species significantly improved soil aggregate stability and organic matter content. Of all the treatments, the T1 treatment exhibited better results, with soil aggregate stability and organic matter content increased to 45.63% and 137.57%, respectively, compared to the control. Soil stability was significant positively correlated with macroaggregate content and negatively with microaggregates. Using structural equation modeling analysis, we further evaluated the mechanism underpinning the influence of organic matter content and fractions on the content of each graded agglomerates. The analysis showed that the macroaggregate content was influenced by the presence of the plant species, primarily realized by altering the content of organic matter and aromatic and amide compounds in the agglomerates, whereas the microaggregate content was influenced by the addition of MSMs, primarily realized by the content of organic matter and polysaccharide compounds. Overall, we observed that the effect of the co-action of MSMs and the plant species was significantly better than that of using MSMs or the plant species alone.

**Discussion:**

The findings of this study provide reliable data and theoretical support for the development and practical application of the APG method to gradually develop and improve the new greening approach.

## 1 Introduction

The utilization of natural resources has greatly contributed to the social and economic progress of mankind (Shao, 2019). However, while providing human and food security, it has resulted in several negative impacts on the environment (Okello et al., 2015; Vanwalleghem et al., 2017; Kattel, 2022), including the formation of a large number of exposed slopes (Wang Y. et al., 2022). Exposed slopes can deteriorate the local microclimate, causing a series of environmental problems, such as landslides and mudslides, and seriously threatening the safety of human life and property (Dyregrov et al., 2018; Newnham et al., 2019; Rosselló et al., 2020). Relying on nature’s power to repair exposed slopes is almost difficult to realize. Therefore, an ever-increasing number of experts and scholars are committed to finding efficient and economical ways to restore the ecology of exposed slopes (Huang et al., 2017; Li et al., 2018; Zhang et al., 2018).

The common technologies for the treatment and restoration of exposed slopes worldwide include the spraying greening method, three-dimensional planting network greening method, vegetation green carpet, planting hole greening, and thick substrate technology (Li et al., 2017; Tang et al., 2018; Li et al., 2019; Liu et al., 2019; Wang Y. et al., 2022). Based on these technologies, a series of studies and engineering practices have been conducted in various countries to continuously optimize the greening substrate and improve engineering technology, and as a result, a variety of new technologies have been gradually developed (Huang et al., 2017; Li et al., 2017; Broda and Gawlowski, 2018; Li et al., 2018; Wang Y. et al., 2022). However, existing technologies still exhibit numerous issues (Tang et al., 2018; Li et al., 2019; Faiz et al., 2022), such as poor adaptability of the substrate on slopes, poor erosion resistance, and ease of spalling. Therefore, improving slope soil stability is key to realizing permanent greening of slopes.

Soil environment reconstruction is the priority of permanent greening of bare slopes (Cao et al., 2015; Han et al., 2019). Soil aggregates are the basic structural units of soil, and their stability is an important indicator of how well the soil performs its coordination mechanisms and environmental functions (Li T. et al., 2020; Guhra et al., 2022). The fragmentation of aggregates has long been recognized as the first and most critical step in the occurrence of soil erosion, which directly affects the stability of soil on slopes, making the study of soil aggregate stability particularly important (Cui et al., 2022; Li et al., 2022; Wang et al., 2023). Nonetheless, there is no unanimous conclusion on the most critical factors affecting the stability of aggregates, but it is widely accepted that the content of soil organic matter is the key to soil aggregate stability (Tisdall and Oades, 1982; Boyle et al., 1989; Obalum et al., 2017; Sarker et al., 2018; Mustafa et al., 2020; Zhou et al., 2020; Voltr et al., 2021).

Microorganisms play an important role in biogeochemical cycles and have great potential for restoring soil function and re-establishing ecological balance (Bertrand et al., 2015). They not only provide nutrients to plants by solubilizing and releasing metal ions from minerals but also change soil nutrient levels through acidolysis and complexation (Khan et al., 2007; Kamran et al., 2017). They can also improve soil conditions, promote plant growth, and increase stress resistance by utilizing microbial metabolism, hormone production, and antagonism (Giassi et al., 2016; Wang et al., 2019; Deng et al., 2020; Abulfaraj and Jalal, 2021). In addition, soil microorganisms secrete and produce organic cementing substances, such as polysaccharides or cement soil clay particles, through mycelia (Yang et al., 2015; Ogarkov et al., 2018; Barbosa et al., 2019; Ji et al., 2019; Chung et al., 2021), thereby promoting the formation of aggregates (Sandhya and Ali, 2015; Deka et al., 2018).

Given the great potential of microorganisms in the revegetation of slopes, we proposed an active permanent greening (APG) method based on selected mineral solubilizing microorganisms (MSMs) (Wang L. et al., 2023). We isolated a variety of microorganisms from the dolomite rock wall of the Mufu Mountains, Nanjing, and selected four typical strains (Jinchi et al., 2014; Guanglin et al., 2015; Jinchi et al., 2015a; Jinchi et al., 2015b) for use in slope management after preliminary experiments on cultivation and solubilization mechanisms. However, studies have been conducted on the effects of MSMs on soil nutrients and plant growth, yet soil aggregates have not been investigated. Therefore, based on the results of previous studies, we selected two superior strains and set up six treatments for controlled experiment. The objectives of the study were (1) to investigate the effects of MSMs and their combination with a plant species on the distribution and stability of soil aggregates, (2) to investigate the effects of MSMs and their combination with the plant species on the content and properties of soil organic matter, and (3) to explore the intrinsic mechanism by which MSMs and their combination with a plant species enhance slope stability by regulating soil aggregation structure.

This study explored the effect of organic matter on soil aggregate stability and its mechanism under the action of microorganisms and plants, and the results will enrich the existing literature on soil stability research. Meanwhile, it demonstrates the role of MSMs in slope management more comprehensively, and helps to promote the systematic research of APG methods, solve the problems related to the traditional greening methods that are difficult to maintain in the long term, and form an efficient, environmentally friendly and sustainable greening technology.

## 2 Materials and methods

### 2.1 Microorganism strains

The strains NL-11 and NL-15 used in this study were isolated from the surface of the weathered rock wall of the Mufu Mountains, Nanjing (rock properties: CaO, 62.34%; MgO, 27.93%; K_2_O, 1.75%; Fe_2_O_3_, 3.00%; Al_2_O_3_, 0.61%; SiO_2_, 1.35%; Na_2_O,0.04%; and others, 2.95%), and screened using adaptation and mineral solubilization tests. These strains were identified using 16S rRNA sequencing as *Bacillus thuringiensis* and *Gongronella butleri* [preserved in the China Center for Type Culture Collection (CCTCC) as M2012453 and M2012454] (Jinchi et al., 2014; Guanglin et al., 2015). These well-preserved strains were inoculated on nutrient agar (peptone, 10.0 g/L; beef extract powder, 3.0 g/L; NaCl, 5.0 g/L; and agar,15.0g/L) and potato sucrose (potato infusion powder, 7.0 g/L; sucrose, 20.0 g/L; and agar, 20.0 g/L) media and cultured at 30°C for 24–48 h. To achieve appropriate colony numbers, cultures of the strains were prepared by inoculating the activated strains individually in nutrient broth (peptone, 10.0 g/L; beef extract powder, 3.0 g/L; and NaCl, 5.0 g/L) and potato liquid (potato dip powder, 6.0 g/L; glucose, 20.0 g/L; and chloramphenicol, 0.1 g/L) media and incubating them at 30°C for 24 h.

### 2.2 Plant material and soil strategies

A pot experiment was conducted by mixing the culture thoroughly with an appropriate amount of sterilized soil (soil properties: effective nitrogen content of 101.88 mg·kg^-1^; effective phosphorus content of 5.44 mg·kg^-1^; effective potassium content of 102.35 mg·kg^-1^; and organic matter content of 7.38 g·kg^-1^). Three replicates were set for each treatment and the initial water content was set at 0.3 cm^3^/cm^3^ (V/V). After six months of routine maintenance, soil samples were collected from the pots for testing, with four sites mixed from each pot.

*Lolium perenne* L., a salt- and drought-tolerant engineered plant, was selected for this study, and its seeds were provided by Heyou Landscaping Engineering Co., Ltd (Jiangsu, China). Six treatment regimens were used in the study (**Table 1**), with sterile water for the control group and three replicates for each treatment.

**Table 1:** Different treatments used in the study.

Treatments	Configuration
**T1**	*B. thuringiensis* (NL-11) + *L. perenne*
**T2**	*B. thuringiensis* (NL-11)
**T3**	*G. butleri* (NL-15) + *L. perenne*
**T4**	*G. butleri* (NL-15)
**T5**	*L. perenne*
**T6**	No microorganisms or plant species (control)

### 2.3 Determination of the distribution and characterization of soil aggregates

Soil samples were collected from three sites for each treatment set-up and mixed. Fresh soil was gently broken along natural cracks to remove plant debris. A certain weight of soil sample was passed sequentially through sieves with pore sizes of 2 mm, 0.25 mm, and 0.053 mm; and weighed individually. Thereafter, the percentage of dry, sieved aggregates at each level was calculated as the percentage of the total amount of soil, and the aggregates were allocated to a certain amount of air-dried soil sample according to the proportion of dry, sieved aggregates. Water-stabilized aggregates of different sizes were then obtained by wet sieving the soil aggregates. Approximately 100 g of prepared soil samples were placed on the top of a set of sieves with pore sizes of 2 mm, 0.25 mm, and 0.053 mm from top to bottom, soaked in distilled water for 5 min, and then shaken vertically up and down for 20 min. The soil samples were transferred to aluminum boxes, dried, and weighed to calculate the weight proportion of each soil aggregate fraction (WSA). Based on the results of agglomerate composition determined by the wet sieving method, the content of water-stable agglomerates with diameter >0.25 mm (R > 0.25 mm), mean weight diameter (MWD), geometric mean diameter (GMD), and fractal dimension (D) were calculated. The formula for each index is as follows (Elliott, 1986; Balestrini et al., 2014):


$$MWD=\sum_{i=1}^{n}x_{i}w_{i}$$

$$GMD=\text{exp}[\sum_{i=1}^{n}w_{i}\times \ln x_{i}]$$

$$R_{>0.25}=\frac{m_{>0.25\;mm}}{m}\times 100\%$$

$$\frac{m_{r<R}}{m_{T}}={\left(\frac{R}{\lambda_{m}}\right)}^{3-D}$$

where, MWD is the mean weight diameter (mm), GMD is the geometric mean diameter (mm), $\;x_{i}$ is the average diameter of the soil particle of any size range (mm), $\;w_{i}$ is the percentage content of aggregates of the i particle size (%), $\;R_{>0.25}$ is the proportion of agglomerates with diameter >0.25 mm (%), $\;m_{>0.25mm}$ is the mass of aggregates with a diameter >0.25mm (g),  m is the mass of water-stable aggregates (g), D is the soil fractal dimension, $m_{r<R}$ is particle size smaller than the grain size R of the soil cumulative weight (g), $m_{T}$ is the total mass of the soil (g), R is the average diameter of the upper and lower limits of the range of particle size (mm), and $\lambda_{m}$ is the upper limit of particle size.

### 2.4 Determination of soil organic matter composition and characterization of soil aggregates

The organic matter content of soil aggregates was determined using the concentrated sulfuric acid–potassium dichromate method (external heating), with a standard solution of ferrous sulfate titrated against an excess of potassium dichromate (Walkley and Black, 1934).

A VERTEX70 Fourier-transform infrared spectrometer (FTIR, Bruker, Hamburg, Germany) was used to analyze the structure of the organic matter in soil aggregates. Briefly, potassium bromide crystals were ground to powder and pressed into tablets after thorough grinding and mixing in the ratio of 1:100 (sample: potassium bromide). The spectra were scanned using an FTIR spectrometer, and the spectra were recorded in the range of 4000–500 cm^-1^, with a resolution of 4 cm^-1^ and 32 scans in the transmission mode (Szymański, 2017; Tafintseva et al., 2019; Lin et al., 2020; Mohamed and Janaki, 2021).

### 2.5 Data analyses

The FTIR spectra were automatically baseline corrected and smoothened using the Omnic 8.2 software, and the absorption peaks were regionally integrated (Lin et al., 2020; Mohamed and Janaki, 2021). The signal intensity of each absorption peak was obtained by calculating the percentage of each absorption peak area to the total peak area. Structural equation modeling (SEM) is a multivariate data analysis method that combines a variety of statistical analyses and is suitable for the study of interrelationships among multiple variables. SEM is utilized to establish, estimate and test causal relationships between indicators. The SPSS 26.0 software was used for statistical analyses. Significant differences between different treatments were determined using one-way analysis of variance (ANOVA) and least significant difference (LSD, *p*< 0.05) test, and the means were compared using Duncan’s test. Data were plotted using the Origin 2021 software.

## 3 Results

### 3.1 Composition and characterization of water-stable agglomerates

**Figure 1:**
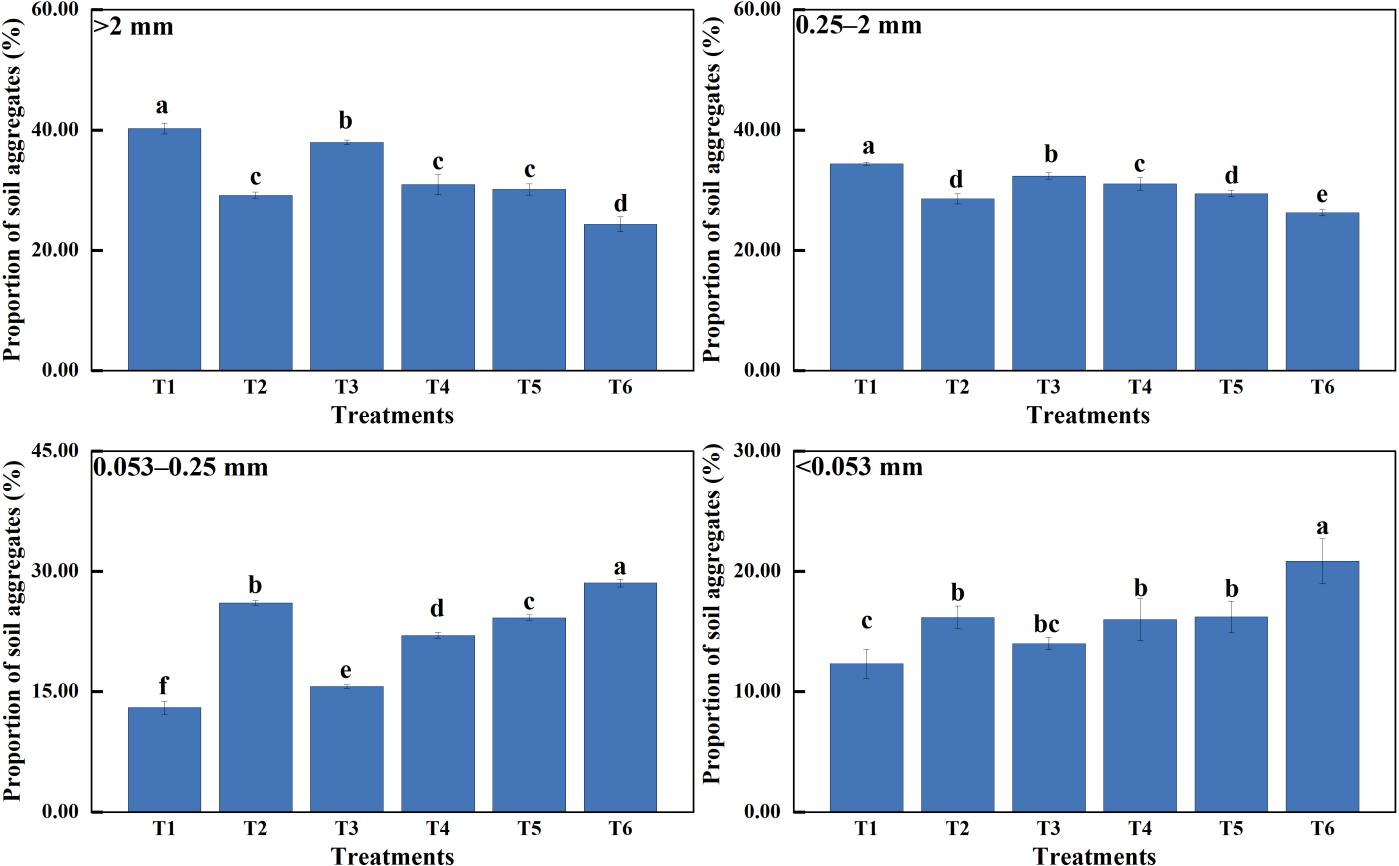
Composition and distribution of water-stable agglomerates. >2 mm: soil aggregates with a diameter >2 mm; 0.25–2 mm: soil aggregates with a diameter of 0.25–2 mm; 0.053–0.25 mm: soil aggregates with a diameter of 0.053–0.25 mm;<0.053 mm: soil aggregates with a diameter<0.053 mm. T1: *B*. *thuringiensis* + *L. perenne*; T2: *B*. *thuringiensis*; T3: *G*. *butleri* + *L. perenne*; T4: *G*. *butleri*; T5: *L. perenne*; T6: Control. Different letters denote significant differences (*P*< 0.05) between various treatments based on one-way analysis of variance and Duncan’s test.

The effect of different treatments on agglomerate composition varied significantly (**Figure 1**). MSMs and plants dramatically increased the weight of agglomerates with diameters of >2 mm and 0.25–2 mm.

The MWD and GWD of soil aggregates varied significantly among treatments (**Table 2**), with a consistent pattern (T1 > T3 > T4 > T5 > T2 > T6), indicating that MSMs and *L. perenne* exhibited a remarkable effect on the stability of soil aggregates. Meanwhile, MSMs and *L. perenne* showed a significant increase in the content of macroaggregates (diameter >0.25 mm) and a decrease of microaggregates (diameter<0.25 mm). The most noticeable effect was observed in the T1 treatment, where R_0.25_, MWD and GWD increased by 47.54%, 45.63% and 84.35%, respectively, compared to the control.

**Table 2:** Characterization of water-stable agglomerates.

	R_0.25_ (%)	MWD (mm)	GMD (mm)	D
**T1**	74.66 ± 0.73 **a**	1.22 ± 0.16 **a**	1.04 ± 0.03 **a**	2.54 ± 0.02 **c**
**T2**	57.74 ± 0.64 **d**	0.95 ± 0.01 **d**	0.70 ± 0.02 **d**	2.59 ± 0.01 **b**
**T3**	70.35 ± 0.54 **b**	1.15 ± 0.01 **b**	0.93 ± 0.02 **b**	2.57 ± 0.01 **bc**
**T4**	61.99 ± 1.55 **c**	1.00 ± 0.03 **c**	0.77 ± 0.05 **c**	2.59 ± 0.02 **b**
**T5**	59.57 ± 1.07 **d**	0.98 ± 0.02 **cd**	0.73 ± 0.03 **cd**	2.59 ± 0.02 **b**
**T6**	50.60 ± 1.43 **e**	0.84 ± 0.03 **e**	0.57 ± 0.03 **e**	2.65 ± 0.02 **a**

R_0.25_, proportion of agglomerates with a diameter >0.25 mm (%); MWD, mean weight diameter (mm); GMD, geometric mean diameter (mm); D, soil fractal dimension. Different letters denote significant differences (P< 0.05) between various treatments based on one-way analysis of variance and Duncan’s test.

### 3.2 Organic matter content and characterization of agglomerates

**Figure 2:**
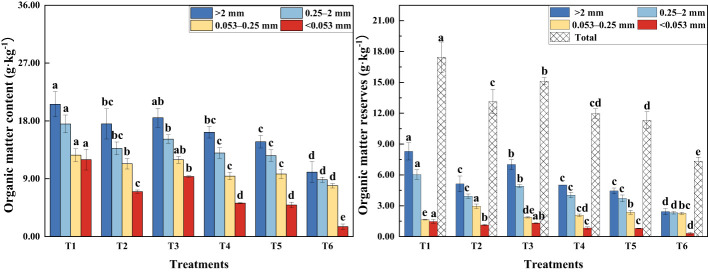
Organic matter content and storage of agglomerates of various grain sizes. >2 mm: soil aggregates with a diameter >2 mm; 0.25–2 mm: soil aggregates with a diameter of 0.25–2 mm; 0.053–0.25 mm: soil aggregates with a diameter of 0.053–0.25 mm;<0.053 mm: soil aggregates with a diameter<0.053 mm. T1: *B*. *thuringiensis* + *L. perenne*; T2: *B*. *thuringiensis*; T3: *G*. *butleri* + *L. perenne*; T4: *G*. *butleri*; T5: *L. perenne*; T6: Control. Different letters denote significant differences (*P*< 0.05) between various treatments based on one-way analysis of variance and Duncan’s test.

The presence of MSMs and the plant species increased the organic matter content of soil aggregates at all grain levels (**Figure 2**). The organic matter content of soil aggregates at different grain levels followed the following order: SOM_>2 mm_ (soil organic matter content of aggregates with a diameter >2 mm) > SOM_2–0.25 mm_ > SOM_0.25–0.053 mm_ > SOM_<0.053 mm_. The organic matter content and storage of T1 sample was significantly higher than the other treatments.

**Figure 3:**
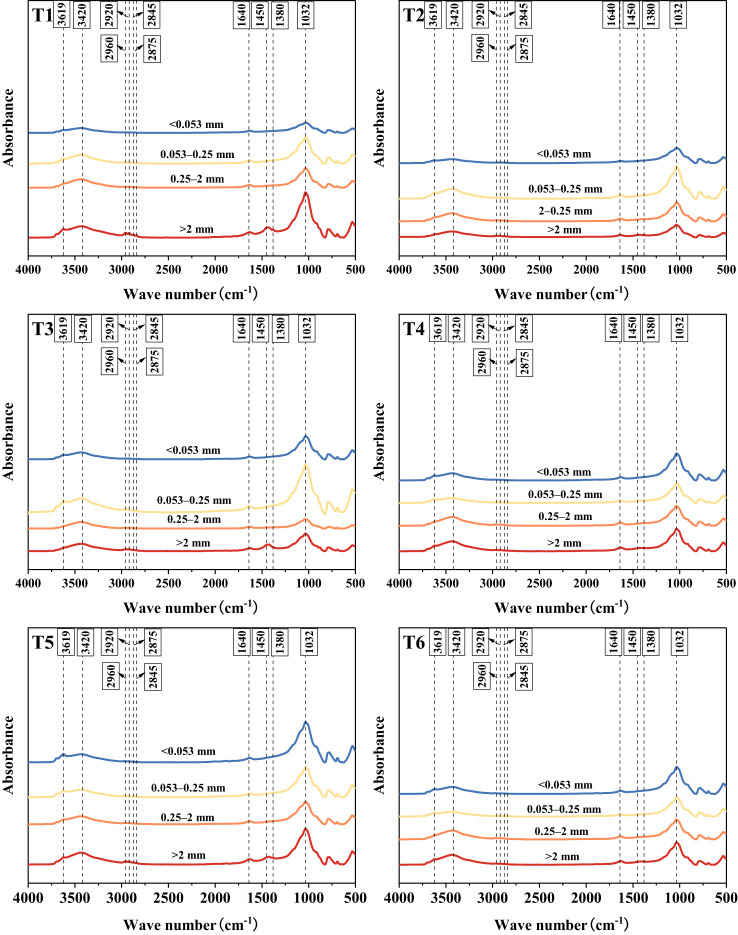
Infrared spectra of agglomerates at each grain level. >2 mm: soil aggregates with a diameter >2 mm; 0.25–2 mm: soil aggregates with a diameter of 0.25–2 mm; 0.053–0.25 mm: soil aggregates with a diameter of 0.053–0.25 mm;<0.053 mm: soil aggregates with a diameter<0.053 mm. T1: *B*. *thuringiensis* + *L. perenne*; T2: *B*. *thuringiensis*; T3: *G*. *butleri* + *L. perenne*; T4: *G*. *butleri*; T5: *L. perenne*; T6: Control.

The infrared spectra of the samples exposed to different treatments and of different grain sizes exhibited similar absorption bands and peaks, revealing the characteristics of polysaccharide, aromatic, aliphatic, amide and alcohol/phenolic functional groups, respectively (**Figure 3**). The different absorption bands and peaks differ only in peak height and peak area, indicating that they exhibited the same functional groups but different contents.

**Figure 4:**
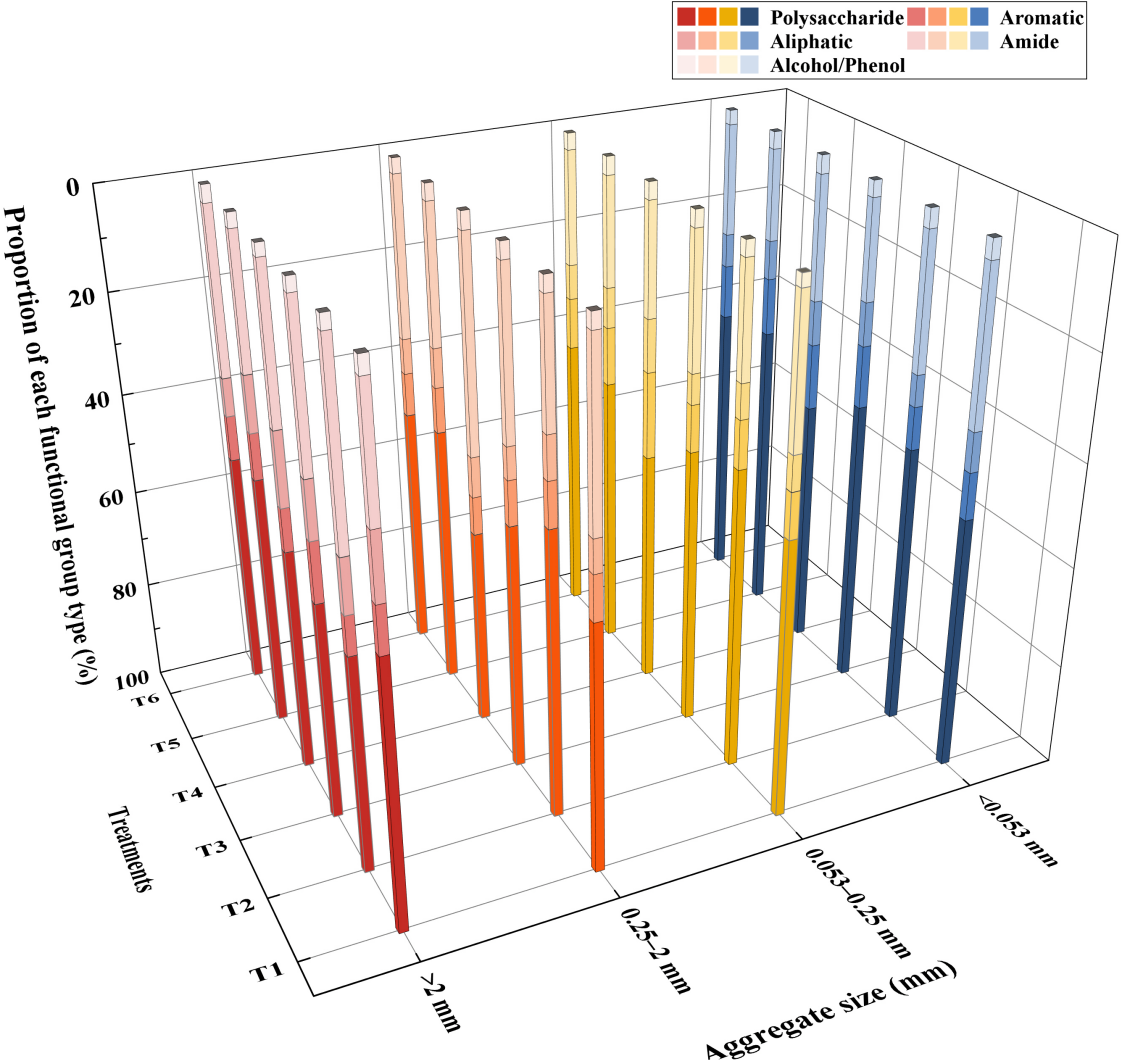
Functional group analysis of agglomerates based on different treatments and aggregate size. >2 mm: soil aggregates with a diameter >2 mm; 0.25–2 mm: soil aggregates with a diameter of 0.25–2 mm; 0.053–0.25 mm: soil aggregates with a diameter of 0.053–0.25 mm;<0.053 mm: soil aggregates with a diameter<0.053 mm. T1: *B*. *thuringiensis* + *L. perenne*; T2: *B*. *thuringiensis*; T3: *G*. *butleri* + *L. perenne*; T4: *G*. *butleri*; T5: *L. perenne*; T6: Control.

The relative contents of major functional groups of different size aggregates in different treatments are shown in **Figure 4**. The highest content of organic matter components in samples exposed to different treatments and of different grain sizes were polysaccharides and amides, followed by aromatic and aliphatic compounds, and the lowest contents were those of alcohols and phenols. For agglomerates with a diameter >2 mm, the presence of MSMs and the plant species increased the content of aliphatic compounds and decreased the content of alcohols and phenols. For agglomerates with a diameter <0.053 mm, the presence of MSMs and the plant species significantly increased the content of alcohols and phenolics.

### 3.3 Correlation analysis of organic matter and water stability characteristics of agglomerates

**Figure 5:**
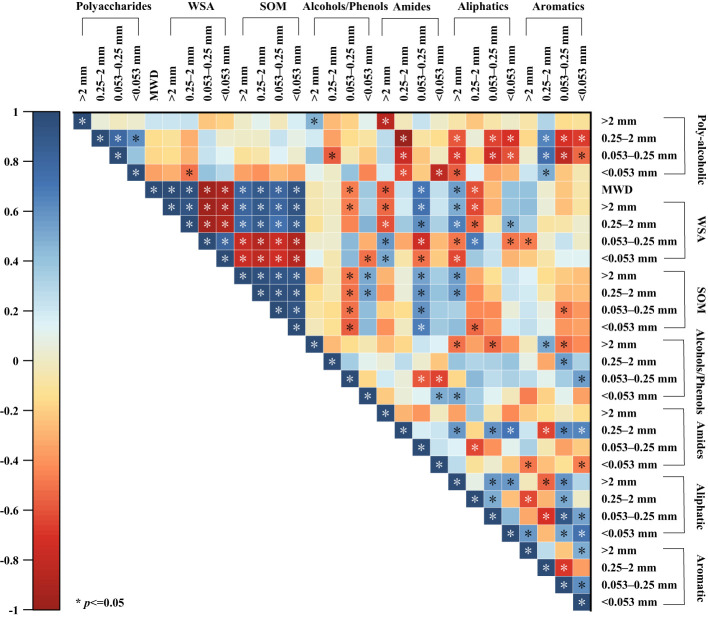
Correlation analysis of organic matter content and water stability characteristics of agglomerates at various grain levels. >2 mm: soil aggregates with a diameter >2 mm; 0.25–2 mm: soil aggregates with a diameter of 0.25–2 mm; 0.053–0.25 mm: soil aggregates with a diameter of 0.053–0.25 mm;<0.053 mm: soil aggregates with a diameter<0.053 mm. * indicates significant differences at *P* ≤ 0.05. SOM: soil organic matter content; WSA: the weight proportion of soil aggregates.

Soil aggregate stability was significantly positively correlated with the weight of aggregates with diameters >2 mm and 2–0.25 mm, whereas it was significantly negatively correlated with the weight of other fractions (diameter of 0.25–0.053 mm, and <0.053 mm) (**Figure 5**). Additionally, soil aggregate stability was positively correlated with the organic matter content of the aggregates of all grades. The weights of aggregates with diameters >2 mm and 2–0.25 mm were positively correlated with their organic matter content, whereas the weights of other graded aggregates were significantly negatively correlated with their organic matter content.

### 3.4 Effects of organic matter composition on the agglomerates of different grain sizes

**Figure 6:**
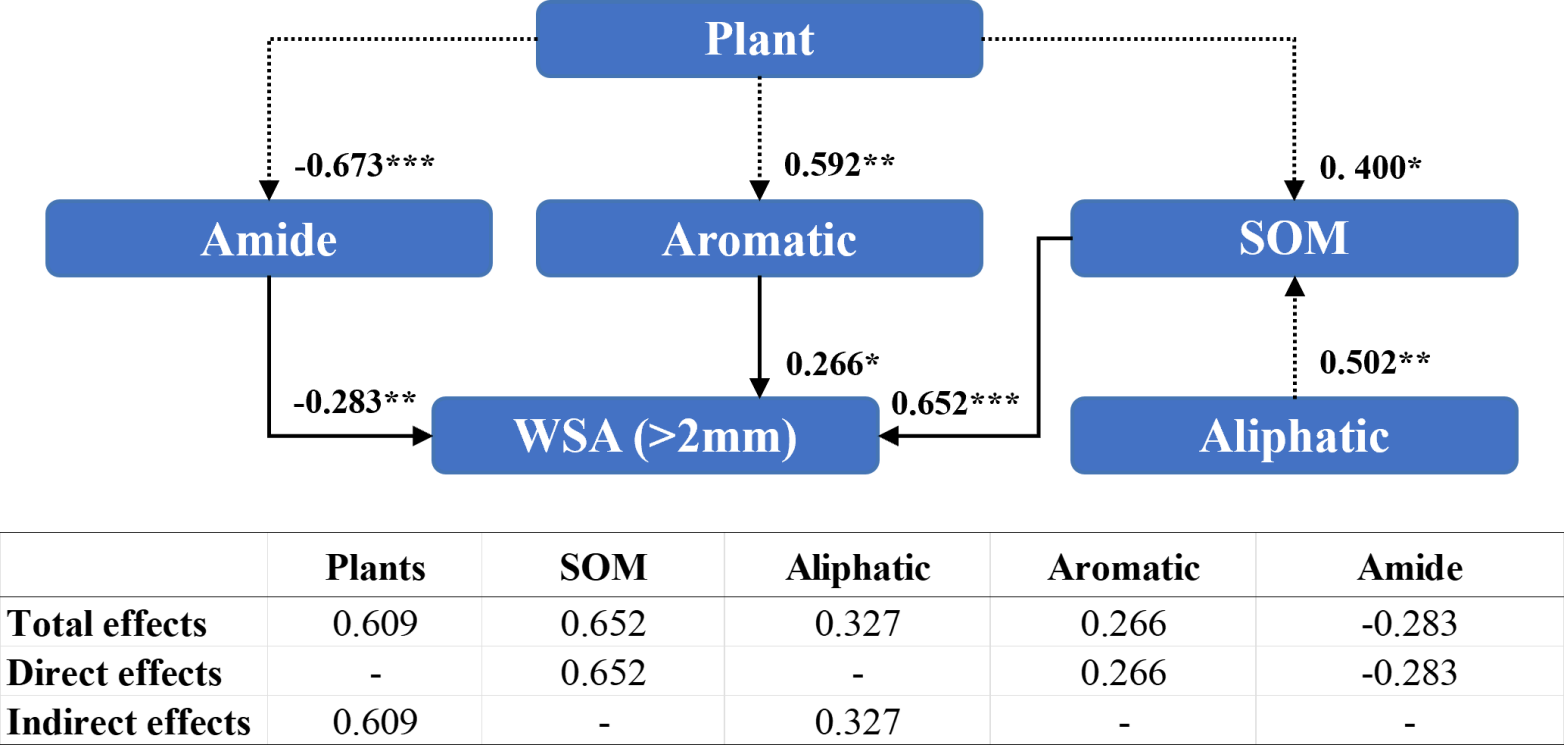
Structural equation modeling of organic matter characteristics affecting the stability of soil aggregates with a diameter >2 mm. CMIN/DF = 1.059; GFI = 0.989; RMSEA< 0.08. Standardized path coefficients are shown as numbers on arrows. ***, **, and * indicate significant differences at *P<* 0.001, *P*< 0.01, and *P*< 0.05, respectively. SOM: soil organic matter content; WSA (>2 mm): the weight proportion of soil aggregates with a diameter >2 mm.

Amides (−0.283), aromatic compounds (0.266), and organic matter content (0.652) directly affected the WSA of diameter >2 mm (**Figure 6**). The presence of the plant species indirectly affected the WSA of diameter >2 mm by altering the content of amide compounds (−0.673), aromatic compounds (0.592), and organic matter content (0.400). The presence of the plant species exhibited a significant positive effect (0.609) on WSA of diameter >2 mm.

**Figure 7:**
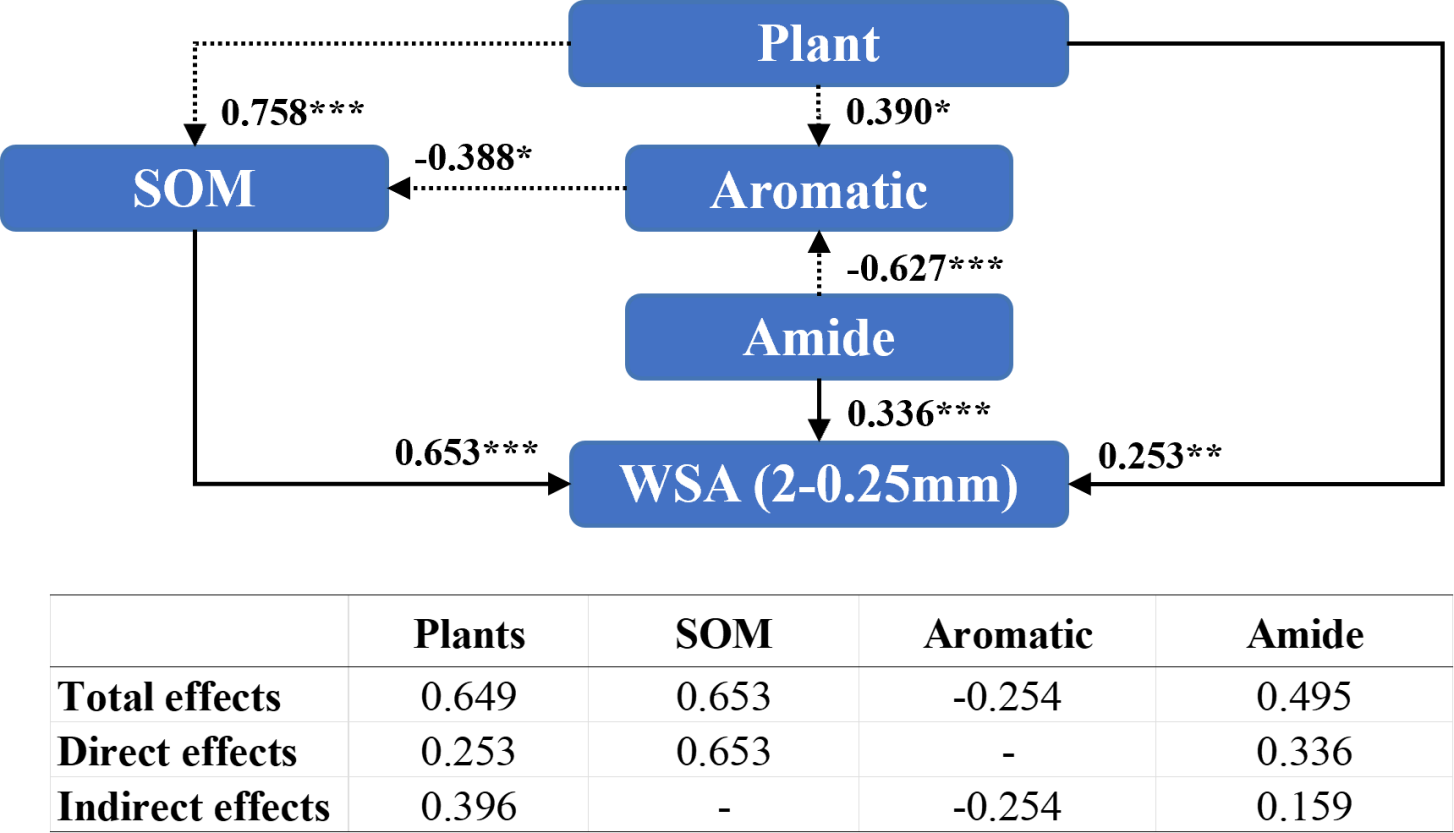
Structural equation modeling of organic matter characteristics affecting the stability of soil aggregates with a diameter between 2 mm and 0.25 mm. CMIN/DF = 1.324; CFI = 0.982; RMSEA< 0.08. Standardized path coefficients are shown as numbers on arrows. ***, **, and * indicate significant differences at *P<* 0.001, *P*< 0.01, and *P*< 0.05, respectively. WSA (2–0.25 mm): the weight proportion of soil aggregates with a diameter between 2 mm and 0.25 mm.

Amide compounds (0.336), organic matter content (0.653) and the plant species (0.253) directly influenced the WSA of diameter between 2 mm and 0.25 mm (**Figure 7**), while the plant species indirectly regulated the WSA of diameter 2–0.25 mm by affecting aromatic compound (0.390) and organic matter contents (0.758). Additionally, the presence of the plant species exhibited a significant positive effect (0.649) on WSA of diameter 2–0.25 mm.

**Figure 8:**
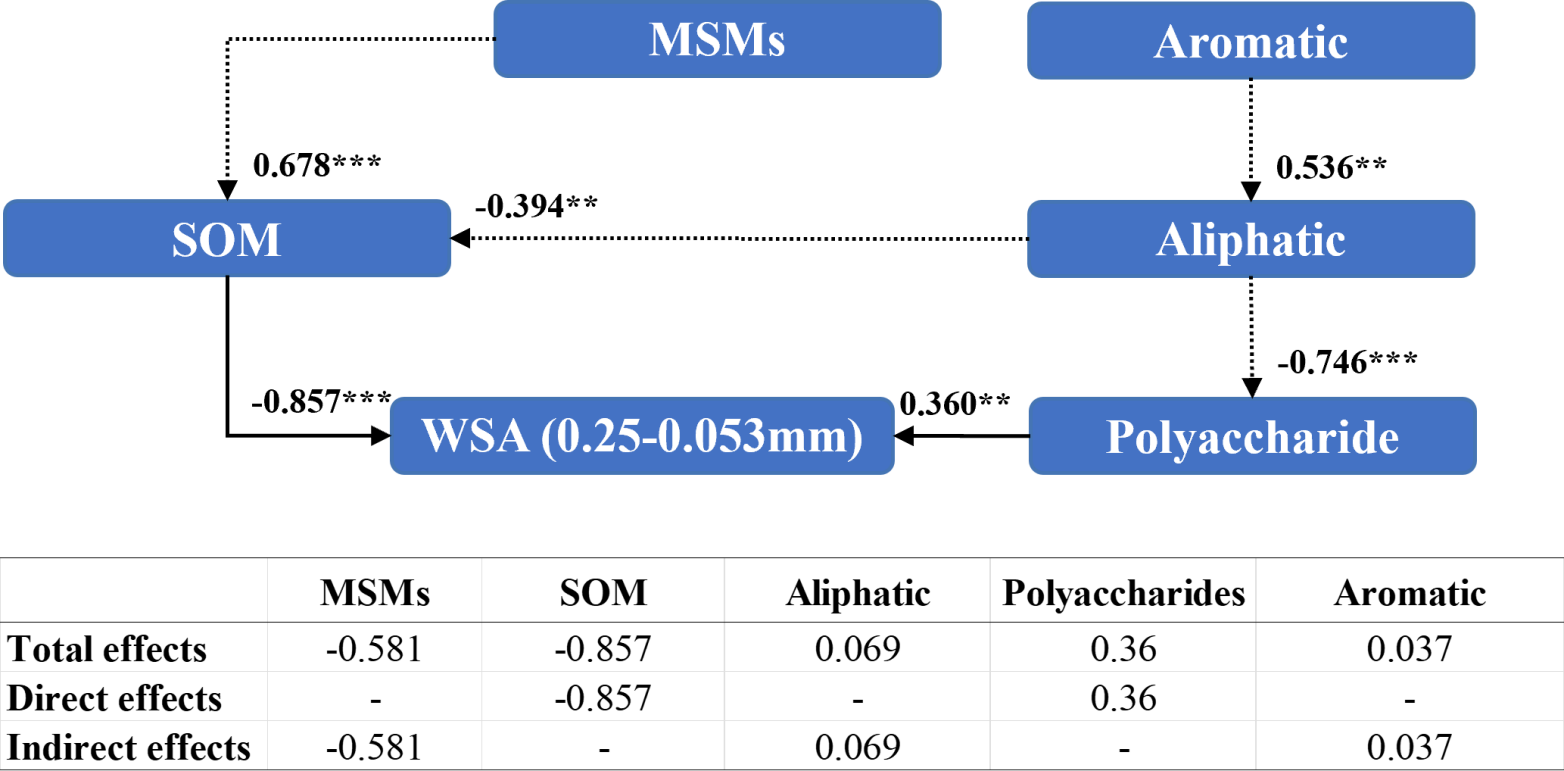
Structural equation modeling of organic matter characteristics affecting the stability of soil aggregates with a diameter between 0.25 mm and 0.053 mm. CMIN/DF = 1.264; CFI = 0.950; MSEA< 0.08. Standardized path coefficients are shown as numbers on arrows. *** and ** indicate significant differences at P< 0.001, P< 0.01, respectively. WSA (0.25–0.053 mm): the weight proportion of soil aggregates with a diameter between 0.25 mm and 0.053 mm

Polysaccharides (0.360) and organic matter content (–0.857) directly affected the WSA of diameter between 0.25 mm and 0.053 mm (**Figure 8**). In contrast, MSMs were able to regulate WSA of diameter 0.05–0.053 mm indirectly by affecting organic matter content (0.678). Nonetheless, the addition of MSMs exhibited a markedly negative influence (–0.581) on WSA of diameter 2–0.25 mm.

**Figure 9:**
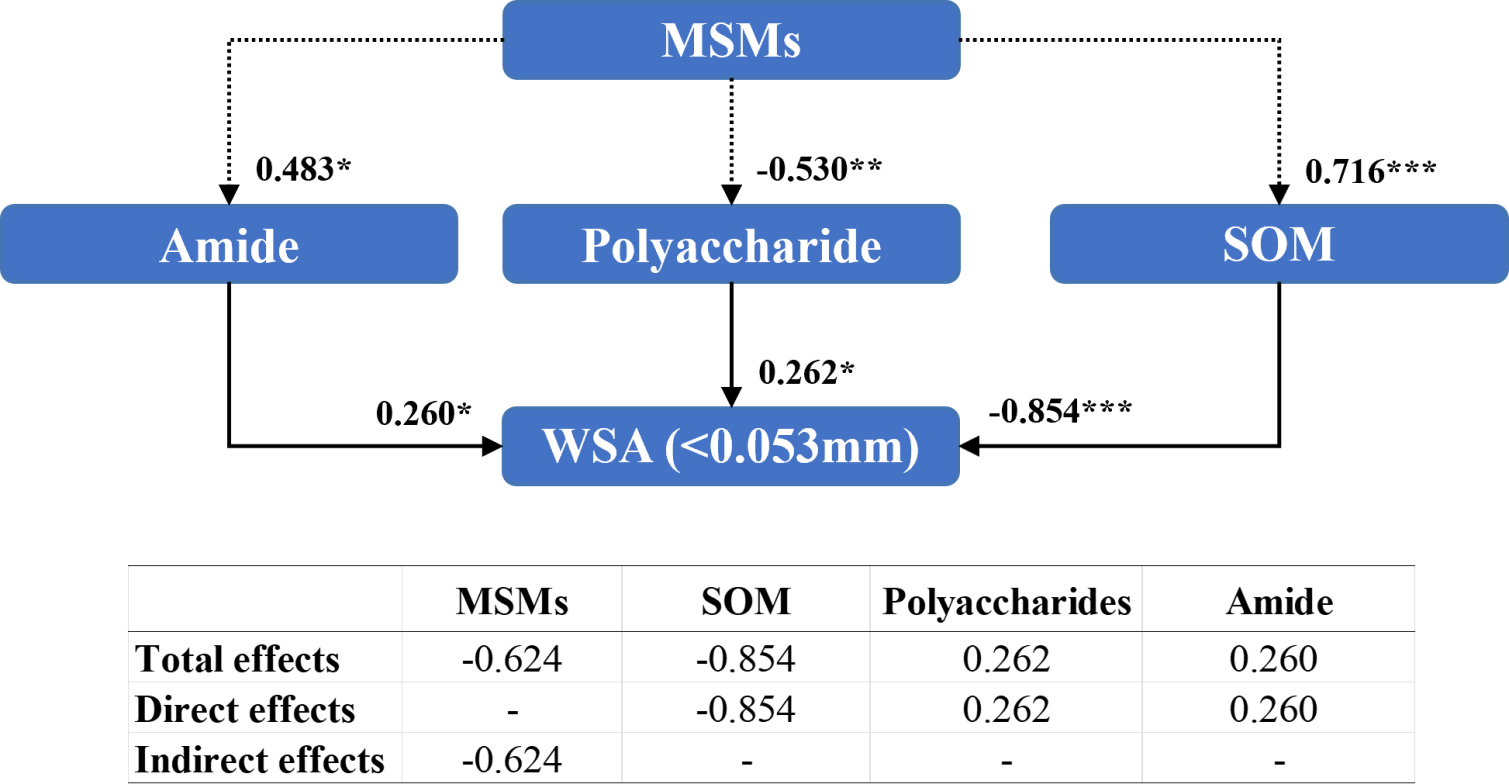
Structural equation modeling of organic matter characteristics affecting the stability of soil aggregates with a diameter between 0.25 mm and 0.053 mm. CMIN/DF = 1.065; CFI = 0.994; RMSEA< 0.08. Standardized path coefficients are shown as numbers on arrows. ***, **, and * indicate significant differences at *P*< 0.001, *P*< 0.01, and *P*< 0.05, respectively. WSA (<0.053 mm): the weight proportion of soil aggregates with a diameter<0.053 mm.

Amides (0.260), polysaccharide compounds (0.262), and organic matter content (-0.854) directly influenced the WSA of diameter<0.053 mm (**Figure 9**). However, MSMs indirectly regulated the WSA of diameter<0.053 mm by affecting polysaccharide (–0.530), amide (0.483), and organic matter contents (0.716). Furthermore, the addition of MSMs exhibited a considerable negative influence (-0.624) on WSA of diameter<0.053 mm.

## 4 Discussion

The rapid population growth in the second half of the last century has intensified the demand for natural resources and interference with nature. At the same time, engineering activities to promote economic development have caused a series of environmental problems (Okello et al., 2015; Vanwalleghem et al., 2017; Kattel, 2022), including the emergence of a large number of bare slopes, and thus ecological and national economic and social development as important constraints cannot be ignored (Rosselló et al., 2020; Wang Y. et al., 2022). Therefore, slope regreening and ecological reconstruction have been the major concerns of researchers (Zhang et al., 2018). Our group has been devoted to theoretical research and technological innovation in slope ecological management and reconstruction for several years by drawing on the experiences and results of slope management measures in various countries and utilizing a series of controlled experiments.

Wu et al. isolated and screened a few microorganisms with strong adaptability and prominent mineral solubilization from the surface of the weathered rock wall of the Mufu Mountains, Nanjing, and explored the mechanisms underlying mineral solubilization (Wu et al., 2017a; Wu et al., 2017b). Jia et al. further investigated the screened microorganisms and found that a variety of MSMs could promote root growth, increase root strength, increase the number of root nodules, and affect the number and species of inter-root microorganisms (Jia et al., 2021). Meanwhile, Li et al. discovered through research that MSMs can affect soil nutrient cycling, especially on the nitrogen cycling with extremely significant effects (Li C. et al., 2020; Li et al., 2021a; Li et al., 2021b). In addition, we conducted a series of optimization studies on slope spraying substrates, including water retention agents (Wang L. et al., 2022), comprehensively analyzed and ranked the effects of different treatments, and preliminarily applied the MSM-based APG method (Wang L. et al., 2023). The results showed that APG was an effective and feasible slope greening method, with great application value and broad application prospects. However, soil restoration and reconstruction are the key to the ecological management of slopes, but the effects of MSMs on soil aggregates using the APG method have not yet been investigated.

Soil microorganisms play an important role in the formation and stabilization of soil aggregates, with bacteria and fungi showing considerable aggregation of soil particles (Deka et al., 2018; Zhao et al., 2018; Hou et al., 2021). It has been shown that *Bacillus* cells and their secretions have remarkable aggregation effects on soil particles, with secretions performing particularly well (Sandhya and Ali, 2015; Ma et al., 2017). Fungi, on the other hand, were not only able to aggregate soil particles through cells and their secretions but were also able to use mycelia to aggregate soil particles and form larger agglomerates through entanglement (Lehmann and Rillig, 2015; Lehmann et al., 2017; Morris et al., 2019; Lehmann et al., 2020). This was consistent with the results of this study, where treatment with MSMs (T2 and T4) showed better structure and stability of soil aggregates compared to the control, and notably, the addition of NL-15 (T4) resulted in higher levels of macroaggregates (diameter >0.25 mm) than the treatments with addition of NL-11 (T2) (**Figure 1**). Plant roots not only influence soil aggregates by releasing various secretions but also rearrange soil particles by root entanglement, thus improving soil aggregate stability (Blankinship et al., 2016; Zeng et al., 2018; Dou et al., 2020; Merino-Martín et al., 2021). This was consistent with the findings of the present study (**Figure 1**). Moreover, MSMs together with the plant species were more effective in enhancing soil aggregate stability than MSMs or the plant species alone. This may be related to the increase in sticky substances in the secretions due to the interaction of MSMs with the plant species (Blankinship et al., 2016; Merino-Martín et al., 2021). It could also be attributed to the fact that MSMs have a significant enhancement effect on the length density of the plant root system, which in turn results in enhanced agglomeration and increased soil stability (Lehmann and Rillig, 2015).

Soil organic matter is fundamental to the soil system and influences soil formation, fertility, soil, and other properties (Murphy, 2015; Obalum et al., 2017; Jin et al., 2020; Voltr et al., 2021). Several studies have shown that the content and composition of soil organic matter depends on both plant inputs and the presence of soil microorganisms (Paul, 2016; Angst et al., 2021). Microorganisms promote the degradation of soil organic matter through decomposition and metabolism to obtain the nutrients they need. Continuous growth and reproduction of microorganisms results in an increase in biomass and the production of large quantities of active substances. They also reduce carbon loss by metabolizing available carbon sources in the soil into more stable organic matter (Kallenbach et al., 2016; Paul, 2016; Liang et al., 2019; Liang et al., 2017). The results of this study showed that the addition of MSMs significantly increased the organic matter content of the soil (**Figure 2**), with a remarkable increase in aliphatic organic matter in macroaggregates (diameter >0.25mm) and alcoholic and phenolic organic matter in microaggregates (diameter<0.25 mm) (**Figure 5**).

The plant root system continuously releases substances or exudates into the soil, including sugars, amino acids, vitamins, long-chain carbohydrates, enzymes, and lysates, during root cell rupturing (Bais et al., 2006; Wang A. et al., 2023). In this study, we noted that the presence of the plant species resulted in a significant increase in the organic matter content of the soil compared to the control (**Figure 2**), especially aliphatic and polysaccharide organic matter in macroaggregates and alcoholic, phenolic, and aliphatic organic matter in microaggregates (**Figure 5**). Simple compounds released in plant root secretions are taken up by microorganisms, attach to mineral surfaces, enter the soil, and eventually form stable organic matter. At the same time, root secretions promote the mineralization of organic matter by stimulating microbial activity and accelerating the decomposition of unprotected carbon in the soil. Total organic matter depends on the net effect of these two opposing mechanisms (Bais et al., 2006; Miltner et al., 2011; Bradford et al., 2013; Cotrufo et al., 2013). In this study, the binding of MSMs to the roots of the plant species significantly increased the organic matter content of the soil (**Figure 2**), especially aliphatic organic matter in macroaggregates and amide organic matter in microaggregates. Notably, the coexistence of MSMs with the plant species (T1 and T3) exhibited improved effects on soil organic matter compared to MSMs alone (T2 and T4). In addition, the promotion of organic matter by NL-11 was more pronounced than that of NL-15, which was also evident by the polysaccharide content in macroaggregates, as well as the amide organic matter content in microaggregates.

Numerous conceptual models regarding the formation and stabilization mechanisms of agglomerates, such as the aggregate hierarchy model (Tisdall and Oades, 1982), aggregate turnover model (Six et al., 2000), and tertiary structure model (Christensen, 2001), have emerged. The results of a large number of studies show that organic matter plays an important role in the process of agglomerate formation, and the higher the content of soil organic matter, the stronger the stability of the formed agglomerates (Boyle et al., 1989; Zhao et al., 2017; Li T. et al., 2020), which is also consistent with the results of the present study (**Figure 5**). In recent years, researchers have focused on the effects of soil organic matter chemistry on soil aggregates (Recio-Vazquez et al., 2014; Guan et al., 2018; Sarker et al., 2018). Oades et al. (Oades and Waters, 1991; Guhra et al., 2022) found that microaggregates are virtually devoid of plant debris and are stabilized primarily by microorganisms and their derivatives, including polysaccharides, microbial cells, and other components. In this study, we observed that the addition of MSMs influenced the organic matter and polysaccharides in the microaggregates and hence the percentage of microaggregate content (**Figures 6**, **7**), whereas in macroaggregates, larger soil particles combined with microaggregates through biological interactions (microbial secretions, root systems, and mycelia) (Six et al., 2000; Rillig et al., 2017). There have been several reports of strong correlation between plant growth and the nature of macroaggregates (Poirier et al., 2017; Schomburg et al., 2018). The present study also noted that the presence of the plant species was vital for macroaggregate content, which could affect the content of organic matter and aromatic compounds and thus the percentage of macroaggregate content (**Figures 8**, **9**).

## 5 Conclusions

To achieve an efficient and cost-effective method for the management of slopes, we proposed the active permanent greening (APG) method based on patented mineral solubilizing microorganisms (MSMs). We investigated the changes in soil aggregates and organic matter using the APG method in a controlled experiment, and explored the intrinsic mechanism by which MSMs and their combination with plants enhance slope stabilization by regulating soil aggregate structure. The results showed that (1) MSMs and the plant species dramatically affected the distribution of soil aggregates and improved their stability. Among them, *G. butleri* performed better and improved soil aggregate stability by 20.72% compared to the control. (2) MSMs and the plant species significantly increased the organic matter content of the soil, where *B. thuringiensis* performed better, improving organic matter content by 78.85% compared to the control. Remarkably, the synergistic effect of MSMs and the plant species on improving soil aggregate stability and organic matter content was better than using MSMs or the plant species alone, with the combination of *B. thuringiensis* and *L. perenne* yielding the best results among all treatments. (3) Macroaggregate (diameter > 2mm) content had a significant positive effect on soil stability, while the other fractions had a significant negative effect. (4) We also used structural equation modeling to investigate the relationship between organic matter and WSA (the weight proportion of soil aggregates) under different combinations of MSMs and the plant species and found that the weight proportion of macroaggregates was mainly affected by the plant species, whereas that of microaggregates was mainly affected by MSMs. The application of the APG method in slope management has great potential and deserves further more in-depth and comprehensive research.

## Patents

Jinchi Zhang, Guanglin Wang, Bo Zhang, Yanwen Wu: An efficient limestone erosion bacterium *Bacillus thuringiensis* NL-11 and its application. CN103087954B; Guanglin Wang, Jinchi Zhang, Jie Lin, Rong Cao: An efficient limestone erosion fungus *Gongronella butleri* NL-15 and its application. CN103087926B.

## Data availability statement

The original contributions presented in the study are included in the article/supplementary material. Further inquiries can be directed to the corresponding author.

## Author contributions

LW: Conceptualization, Data curation, Formal Analysis, Investigation, Methodology, Software, Validation, Writing – original draft. XT: Investigation, Methodology, Software, Validation, Writing – review & editing. XL: Investigation, Methodology, Project administration, Software, Supervision, Validation, Writing – review & editing. RX: Data curation, Investigation, Methodology, Software, Writing – review & editing. JZ: Formal Analysis, Funding acquisition, Project administration, Resources, Writing – review & editing.
